# Digital Interventions to Enhance Readiness for Psychological Therapy: Scoping Review

**DOI:** 10.2196/37851

**Published:** 2022-08-30

**Authors:** Jacinta Jardine, Robert Bowman, Gavin Doherty

**Affiliations:** 1 School of Computer Science and Statistics Trinity College Dublin Dublin Ireland

**Keywords:** readiness for change, stages of change, digital, motivation, engagement, uptake, mental health, mental illness, mobile phone

## Abstract

**Background:**

Psychological therapy is an effective treatment method for mental illness; however, many people with mental illness do not seek treatment or drop out of treatment early. Increasing client uptake and engagement in therapy is key to addressing the escalating global problem of mental illness. Attitudinal barriers, such as a lack of motivation, are a leading cause of low engagement in therapy. Digital interventions to increase motivation and readiness for change hold promise as accessible and scalable solutions; however, little is known about the range of interventions being used and their feasibility as a means to increase engagement with therapy.

**Objective:**

This review aimed to define the emerging field of digital interventions to enhance readiness for psychological therapy and detect gaps in the literature.

**Methods:**

A literature search was conducted in PubMed, PsycINFO, PsycARTICLES, Scopus, Embase, ACM Guide to Computing Literature, and IEEE Xplore Digital Library from January 1, 2006, to November 30, 2021. The PRISMA-ScR (Preferred Reporting Items for Systematic Reviews and Meta-Analyses extension for Scoping Reviews) methodology was applied. Publications were included when they concerned a digitally delivered intervention, a specific target of which was enhancing engagement with further psychological treatment, and when this intervention occurred before the target psychological treatment.

**Results:**

A total of 45 publications met the inclusion criteria. The conditions included depression, unspecified general mental health, comorbid anxiety and depression, smoking, eating disorders, suicide, social anxiety, substance use, gambling, and psychosis. Almost half of the interventions (22/48, 46%) were web-based programs; the other formats included screening tools, videos, apps, and websites. The components of the interventions included psychoeducation, symptom assessment and feedback, information on treatment options and referrals, client testimonials, expectation management, and pro-con lists. Regarding feasibility, of the 16 controlled studies, 7 (44%) measuring actual behavior or action showed evidence of intervention effectiveness compared with controls, 7 (44%) found no differences, and 2 (12%) indicated worse behavioral outcomes. In general, the outcomes were mixed and inconclusive owing to variations in trial designs, control types, and outcome measures.

**Conclusions:**

Digital interventions to enhance readiness for psychological therapy are broad and varied. Although these easily accessible digital approaches show potential as a means of preparing people for therapy, they are not without risks. The complex nature of stigma, motivation, and individual emotional responses toward engaging in treatment for mental health difficulties suggests that a careful approach is needed when developing and evaluating digital readiness interventions. Further qualitative, naturalistic, and longitudinal research is needed to deepen our knowledge in this area.

## Introduction

### Background

Mental illness is a pervasive global problem, estimated to be the second most prominent cause of the global burden of disease, surpassed only by cardiovascular disease [1]. Psychological therapy is both an effective and acceptable treatment for common mental illnesses such as anxiety and depression [2,3], with comparable outcomes across all approaches (ie, cognitive behavioral therapy [CBT], psychodynamic, and person centered) [4] and delivery formats (ie, face-to-face [FTF] and digital format) [5]. Despite the demonstrated effectiveness of psychological therapy, there remains an alarming difference between the number of people with a mental illness and the number of people being treated, often referred to as the *mental health treatment gap* [6]. This gap is substantial and ever expanding, as prevalence rises without a corresponding rise in treatment outreach or provision [6].

One significant problem that perpetuates this treatment gap is *client engagement* in therapy [7,8]. Engagement is a term with many associated meanings [9,10]; for the purposes of this review, we use it as an overarching term to represent client uptake (ie, whether the client begins treatment), as well as the client’s ongoing, active participation in treatment. Client engagement is essential for clients to obtain said favorable outcomes [11-13]; however, because it is an internal cognitive state, it is difficult to measure [9]. Consequently, engagement is often inferred from observing more easily quantifiable metrics, such as adherence, dropout, and use in the digital realm. The uptake rates of digital mental health treatments are estimated to range from 3% to 25% [14]. Low use and high dropout rates are persistent problems when it comes to digital solutions [15], although similar problems also affect FTF modalities; between 17% and 25% of clients are estimated to drop out of FTF psychotherapy [8,16,17]. Considering that only 20% of the people with mental health problems seek treatment in the first place [18], the problem becomes even more apparent. Therefore, increasing client engagement is a key focus area in the wider mental health sphere [10].

### Barriers to Engagement

Many of the practical barriers that have historically impeded access to and engagement with FTF psychological therapy (eg, cost, accessibility, and time constraints) [19] have been reduced with the emergence of digitally delivered treatments. However, this new treatment modality introduces its own set of novel barriers, such as internet anxiety, privacy concerns, lack of confidence in using technology, and disbelief in the effectiveness of the treatments themselves [20-22].

Arguably, the most significant barriers to engagement across all types of therapy delivery stem from the client’s attitude toward seeking help and engaging in therapy [23]. Among these attitudinal barriers, low perceived need, a preference to deal with the problem on one’s own, and internalized self-stigma are the most common [21,23,24].

### Motivation to Change

Motivation is a term used to describe the analytical and habitual processes that energize and direct behavior [25], thus encompassing the attitudinal barriers discussed earlier, among other factors. It is easy to assume that individuals presenting for treatment are motivated to engage in the process and make changes in their lives; however, research indicates that up to 80% of the people who seek treatment are not ready to change and that a leading cause of treatment dropout and low adherence is a lack of motivation [26,27].

The most prominent theory explaining motivation for therapy and readiness for change is the *Transtheoretical Model* (TTM), which posits that clients move through a series of stages on their journey toward and through the process of change [28]. This theory describes behavior change not only in terms of action but also as a wider contemplative process that begins before a person is even considering change [17]. The *stages of change* presented in the TTM are precontemplation, contemplation, preparation, action, and maintenance [28]; the stage a client is in before treatment positively correlates with their outcomes after treatment (ie, the further along they are in terms of the stages, the better their outcomes) [29,30].

The mechanism by which a client’s stage of change affects their overall therapy outcomes could manifest in their initial experiences of treatment [31]. For example, if the client is in the action stage at the onset of treatment, they can fully engage with the process immediately rather than spending initial sessions or interactions in ambivalence, thus delaying improvements [32-34]. As symptom changes that occur early in treatment are linked to greater overall treatment success [11,33], targeting those clients who are not yet in the action stage of change *before* they commence treatment, with interventions designed to move them toward action, could mean that more clients begin therapy, stay engaged, and reach positive outcomes.

### Motivational Interviewing

There are several FTF pretherapy interventions aimed at moving clients through the stages of change and preparing them for subsequent therapy. Examples of such pretherapy interventions include motivational interviewing (MI), role induction, and vicarious therapy pretraining [27]. MI is arguably the most established of these interventions due to its significant effects on client adherence to subsequent therapy as well as treatment outcomes [26,32,35]. MI is a collaborative, discursive therapeutic approach that aims to guide rather than direct clients, fostering autonomy through open questions and evoking the client’s personal reasons for change [36,37]. The specific techniques or tools used by MI practitioners (we will refer to these as “components”) include exploring reasons for change, weighing up the pros and cons of change, developing discrepancy between the client’s ideal and current states, and building confidence and self-efficacy [38]. The key causal model of MI is that client speech affects client outcome [39], meaning that the more favorably a client talks about behavior change, the more likely they are to make the change. Helping clients achieve this “change talk” is a highly nuanced, conversational art undertaken by skilled practitioners over multiple sessions [36]. Originally developed as a brief stand-alone intervention for alcohol misuse, MI is now used as a pretherapy intervention for a range of mental illnesses, including anxiety and depression [35]. However, owing to its current FTF delivery format, traditional MI is not a widely available or accessible option for millions of people experiencing mental health difficulties around the world. Finding a feasible way to deliver interventions, such as MI, in a more accessible format could help more clients become motivated and begin treatment ready to take action.

### Digital Readiness Interventions

Digital methods of intervention delivery hold promise as a way of creating accessible and timely solutions that can be easily scaled to cover entire populations, including those who have not yet reached out for help [10]. A recent systematic review of technology-assisted MI indicated its potential in this area [38]. However, in most of the included studies, the MI components were integrated with other approaches (eg, CBT) and used as stand-alone digital treatments targeted at changing problem behaviors, such as alcohol use and smoking [38], rather than as motivational *pretherapy* interventions. The extent to which digitally delivered MI has been used as a readiness intervention to prepare clients for therapy is unclear. Furthermore, little is known about the feasibility of delivering such a conversational and highly tailored process via digital means [38].

Outside MI, other digital approaches have begun to emerge, such as engagement-facilitation interventions, which aim to increase both the uptake of and adherence to web-based mental health programs [40]. The components of these interventions differ from those used in MI; for example, engagement-facilitation interventions include components such as expectation setting, psychoeducation about symptoms and treatment, treatment belief enhancement, symptom assessment, and assessment feedback [20]. At present, little is known about the full range of different types of digital interventions that are being used to prepare clients for further therapy, the components of these interventions, or the design processes used in their development. Furthermore, research in this area is spread across the digital health, behavior change, and human-computer interaction fields. Thus, a review is needed to scope this topic and clarify the current dispersed and diverse body of research.

Collaboration with clinical professionals and human-centered design processes are key to developing effective mental health interventions, given their sensitive and complex nature [41,42]. As this is an emerging field, formative research exploring intervention design, development, and evaluation can provide insights into opportunities, barriers, and design strategies that can be used to create effective and acceptable solutions.

### This Study

The aim of this study was to define the emerging field of digital interventions to enhance readiness for psychological therapy. By exploring the current state of research in this area, we hope to identify the conceptual boundaries of the topic and identify gaps in the literature. Our research questions were as follows:

1. What types of digital interventions have been used to prepare clients for psychological therapy?
2. What components have been used in these interventions and which of these show evidence of effectiveness?
3. What design processes have been used to develop these interventions?
4. Is the digital delivery of preparatory interventions to enhance readiness for psychological treatment feasible?

## Methods

### Protocol and Structure

The protocol for this review was registered with the Open Science Framework on March 26, 2021 [43]. We used the PRISMA-ScR (Preferred Reporting Items for Systematic Reviews and Meta-Analyses for Scoping Reviews) guidelines to structure our review [44].

### Study Design

We chose a scoping review approach because the research studies in question are heterogeneous in nature and spread across multiple disciplines; they use different study designs to measure different outcomes, with different populations, in different contexts. As this is an emerging field, there are few boundaries on the extent, range, and nature of evidence [44], and the terminology used in the published literature is inconsistent and varied [45]. Therefore, this exploratory review type is well suited.

### Eligibility Criteria

Publications were included for assessment if they met the following criteria: (1) the article concerns an intervention, a specific target of which is enhancing engagement with *further psychological* treatment or therapy; (2) the intervention is delivered *digitally* (ie, the primary active content of the intervention is digital), but studies that use technology solely as a means of synchronous communication (eg, web chat or video calls) were excluded; (3) the intervention took place *before* the target psychological treatment (ie, not combined or performed in tandem with the target treatment); (4) the article was written in English; (5) the article was published in a peer-reviewed publication between 2006 and 2021; and (6) the intervention was designed for adult or adolescent populations (ie, age ≥12 years).

The rationale for examining only recent evidence (past 15 years) is that digital technology is advancing rapidly; older studies may be out of date in terms of client attitudes and acceptance of technology [46]. Comparable time frames have been used in many recent reviews on digital mental health technologies [38,46,47]. We included adolescent populations in our review because research indicates that the main barriers to engagement with mental health treatments are comparable across adult and adolescent populations [48].

### Search Strategy

The following electronic databases were searched: PubMed, PsycINFO, PsycARTICLES, Scopus, Embase, ACM Guide to Computing Literature, and IEEE Xplore Digital Library. Search terms reflected the 3 main eligibility criteria (Table 1).

**Table 1:** Search terms.

Criteria	MeSH^a^ terms	Free-text terms
Target treatment (further psychological treatment or therapy)	“Mental Health” OR “Psychotherapy” OR “Stress, Psychological” OR “Anxiety Disorders” OR “Mood Disorders”	“CBT” OR “psychological” OR “mental ill-health” OR “anxiety” OR “depressi*” OR “stress” OR “wellbeing” OR “well-being” OR “resilience” OR “mood” OR “disorder*” OR “phobia*”
Digital delivery	“Therapy, Computer-Assisted” OR “Internet” OR “Digital Technology”	“digital” OR “technolog*” OR “comput*” OR “e-health” OR “ehealth” OR “m-health” OR “mhealth” OR “mobile” OR “online” OR “web” OR “web-based” OR “smartphone*”
Intervention type (readiness intervention; takes place before the target treatment)	“Transtheoretical Model” OR “Motivational Interviewing”	“readiness” OR “pre-therapy” OR “pre-treatment” OR prepar* OR “prelude” OR “prequel” OR “prior” OR “stage of change” OR “stages of change” OR “motivation to change” OR “motivational enhancement” OR “motivation interview” OR “motivational intervention”

^a^MeSH: Medical Subject Headings.

### Data Collection

An initial exploratory search of PubMed and ACM databases was conducted, and words contained in the titles and abstracts of retrieved papers were analyzed. The search terms were adjusted based on the identified papers, and the final search strategy was decided. Once the protocol was registered with the Open Science Framework, a full search was undertaken across all included databases in March 2021; the search was updated in November 2021. Additional records were retrieved by checking the reference lists of included articles.

The first and second authors (JJ and RB) began by independently reviewing a subset (1300/9412, 15%) of the titles and abstracts against the eligibility criteria and comparing their findings. Discrepancies were found; hence, the eligibility criteria were clarified through discussion between the 2 authors by using relevant examples from the first sample reviewed. A further subset (1300/9412, 15%) was reviewed, the findings were compared, and consensus in decision-making about inclusion and exclusion was reached. The remaining articles were then split between the 2 reviewers (JJ and RB), who independently assessed the titles and abstracts. The final list of selected articles was reviewed by both reviewers. The first author (JJ) then retrieved the full text of the selected articles, and both reviewers independently evaluated them against the eligibility criteria. Reasons for exclusion were recorded, and where there were discrepancies, a discussion was held between the 2 reviewers, and a consensus was reached on the final selection of articles.

### Data Analysis

Data charting was performed in Excel (Microsoft Corporation) by the first author (JJ), with checks and calibration by the second author (RB). The data charting form contained general study details as well as variables related to research questions, including target treatment, intervention type (eg, technology used, duration, and interaction level), intervention pathway (eg, how and when the intervention was delivered to clients and the relationship between the intervention and target treatment), intervention components, the model or framework used, measures and outcomes, user experience or acceptability, design process, critical appraisal (eg, limitations in the study, biases, strength of methodology, and generalizability of results), and key learnings.

### Synthesis of Results

The charted data were further summarized based on the key characteristics of the data. For example, within a charted column such as *the target treatment* or *duration*, findings were assessed in relation to each other, and overarching categories were created based on the most common results. The frequency of occurrences was then examined, and result tables were created. In terms of more complex findings, such as components and outcomes, separate Excel worksheets were created, where individual studies or interventions could be explored in more detail. Frequent checks of the full paper were conducted to validate the initial charting.

## Results

### Study Selection

**Figure 1:**
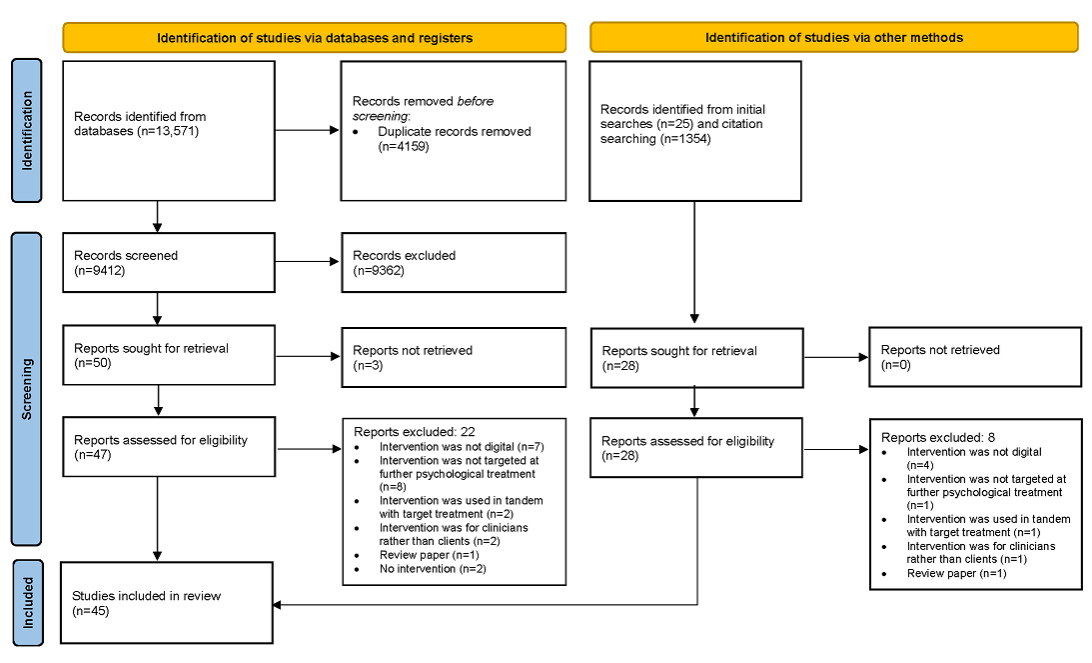
Flow diagram of the study selection process.

The search resulted in 13,571 hits. A further 1379 studies were identified via other sources. After removing duplicates and screening titles, abstracts, and full texts, 45 (0.30%) papers met the eligibility criteria (Figure 1).

### Study Characteristics

The studies included in this review (Table 2) were mainly conducted in the United States (17/45, 38%), Australia (9/45, 20%), and Germany (7/45, 15%). Only 2% (1/45) of the studies included multiple countries [49]. In terms of study design, among the 45 studies, 19 (43%) were randomized controlled trials, 7 (16%) were observational studies, 6 (13%) were protocols, 4 (9%) were studies exploring the development process of interventions, 3 (7%) were pre-post designs, 2 (4%) were nonrandomized controlled trials, 2 (4%) were historically controlled studies, 1 (2%) was a qualitative evaluation, and 1 (2%) study presented the results of multiple studies (2 randomized controlled trials and 1 pre-post study). Only 26% (12/45) of the studies included qualitative data collection, 18% (8/45) used mixed methods [50-57], and 9% (4/45) were purely qualitative [58-61]. Depression (9/45, 20%) and unspecified general mental health (9/45, 20%) were the most common target conditions covered, with comorbid anxiety and depression being the next most frequent (6/45, 14%). The other conditions and problems targeted included smoking (5/45, 11%), eating disorders (5/45, 11%), suicide (3/45, 7%), social anxiety (3/45, 7%), substance use (2/45, 4%), gambling (2/45, 4%), and psychosis (1/45, 2%).

**Table 2:** Characteristics of the included papers.

Author, study, and year	Study design	Country	Condition
Christensen et al [62], 2006	RCT^a^	Australia	Depression
Reis and Brown [63], 2006	RCT	The United States	General mental health
Haas et al [57], 2008	Observational	The United States	Suicide
Costin et al [64], 2009	RCT	Australia	Depression
Olson et al [56], 2009	Historically controlled	The United States	General mental health
Titov et al [65], 2010	RCT	Australia	Social anxiety
Brunette et al [66], 2011	NRCT^b^	The United States	Smoking
Johansen et al [67], 2011	RCT	The United States	General mental health
Strassle et al [68], 2011	RCT	The United States	General mental health
Ferron et al [69], 2012	Observational	The United States	Smoking
Reins et al [70], 2013	Protocol	Germany	Depression
Hötzel et al [71], 2014	RCT	Germany	Eating disorder
Taylor-Rodgers and Batterham [72], 2014	RCT	Australia	Anxiety and depression
Ahmedani et al [73], 2015	Pre-post	The United States	Depression
Ebert et al [14], 2015	RCT	Germany	Depression
King et al [74], 2015	RCT	The United States	Suicide
Batterham et al [75], 2016	RCT	Australia	Anxiety and depression
BinDhim et al [49], 2016	Observational	Australia, the United Kingdom, Canada, New Zealand, and the United States	Depression
Moessner et al [76], 2016	Observational	Germany	Eating disorder
Birnbaum et al [77], 2017	Observational	The United States	Psychosis
Bommelé et al [78], 2017	NRCT	The Netherlands	Smoking
Brown et al [55], 2017	Development process	The United States	Smoking
Griffiths et al [79], 2017	RCT	Australia	Social anxiety
Krampe et al [80], 2017	RCT	Germany	General mental health
Metz et al [81], 2017	Protocol	The Netherlands	General mental health
Muir et al [58], 2017	Development process	The United Kingdom	Eating disorder
Liu et al [60], 2018	Development process	New Zealand	General mental health
Suka et al [82], 2018	Observational	Japan	Depression
Batterham et al [40], 2019	Protocol	Australia	Anxiety and depression
Dannenberg et al [59], 2019	Development process	The United States	Depression
Denison-Day et al [54], 2019	RCT	The United Kingdom	Eating disorder
Dreier et al [50], 2019	Protocol	Germany	Suicide
Ebert et al [83], 2019	RCT	Germany	General mental health
Johansen et al [61], 2019	Qualitative	Norway	Gambling
McLean et al [84], 2019	Observational	Australia	Eating disorder
Shand et al [85], 2019	Protocol	Australia	Depression
Beck et al [51], 2020	Pre-post	Canada	Anxiety and depression
Brunette et al [86], 2020	RCT	The United States	Smoking
Duffy et al [53], 2020	Pre-post	The United Kingdom	Anxiety and depression
Peter et al [87], 2020	RCT	The United States	Gambling
Keller et al [52], 2021	RCT and pre-post	The United States	General mental health
Olthof et al [88], 2021	Protocol	The Netherlands	Substance use
Soucy et al [89], 2021	RCT	Canada	Anxiety and depression
Tobias et al [90], 2021	RCT	The United States	Social anxiety
Yoon et al [91], 2021	Historically controlled	The United States	Substance use

^a^RCT: randomized controlled trial.

^b^NRCT: nonrandomized controlled trial.

### Types of Intervention

To assess the interventions analyzed in the included papers, we first distinguished the interventions themselves from the papers. A total of 6 studies in the sample [62,64,65,67,86,90] assessed 2 distinctly different interventions in their analysis (ie, the components of the interventions were distinct); therefore, we separated them into individual records. In all, 2 studies assessed slight variations in the same intervention [52,87]; however, we did not segregate these studies because they only reflected minor variations in what was essentially the same intervention. Furthermore, 3 sets of 2 studies in the sample analyzed the same interventions: [66,69], [54,58], and [51,89]; therefore, we grouped them together. The final list of 48 interventions is analyzed in this section and the subsequent one.

We explored the interventions under several categories: intervention format, target treatment or therapy for which the intervention was designed to prepare clients for, the level of support provided, whether the intervention was designed for repeated or once-off use, the duration of the intervention, the theoretical model used, and how the intervention was delivered to the client (Table 3). We found many variations in the types of interventions used to prepare people for psychological therapy. Almost half of the interventions (22/48, 46%) were web-based programs; the other formats included screening tools, videos, apps, and websites. Many of the included interventions were not designed to prepare clients for a specific treatment but instead to encourage general professional help seeking (27/48, 56%). Of those targeted at specific treatments, FTF therapy was the most common (14/48, 29%), followed by web-based therapy (6/48, 13%), and phone therapy (1/48, 2%). In terms of the duration of the interventions, those that specified a duration ranged from 15 seconds to 6 months, with most interventions taking <90 minutes to complete (23/48, 48%). We also investigated how and when the interventions were delivered to clients. Most of the interventions (32/48, 67%) were delivered to clients who had not already sought help via outreach methods such as social media, marketing, or email. Excluding a study that was unclear, the remaining 15 (31%) interventions were delivered to clients who had already sought help or were investigating help.

**Table 3:** Types of interventions in the selected studies (N=48).

Category				Studies, n (%)
**Intervention format**				
	Web-based program		22 (46)	
	Screening tool		7 (15)	
	Video		6 (13)	
	App		4 (8)	
	Website		3 (6)	
	Automated emails and website		2 (4)	
	Screening tool and messaging		2 (4)	
	Advertisement		1 (2)	
	Advertisement and website		1 (2)	
**Target treatments**				
	General professional help		27 (56)	
	**Specific treatments**		21 (44)	
		Specific face-to-face therapy	14 (29)	
		Specific web-based therapy	6 (13)	
		Specific phone therapy	1 (2)	
**Support**				
	No support		35 (73)	
	**Supported**		13 (27)	
		Asynchronous (clinician)	5 (10)	
		Synchronous (digital)	4 (8)	
		Synchronous (clinician)	2 (4)	
		Asynchronous and synchronous (peer)	1 (2)	
		Asynchronous and synchronous (clinician)	1 (2)	
**Use**				
	Once-off		28 (58)	
	Repeated		20 (42)	
**Intervention duration (estimated or average)**				
	**Duration (minutes or hours)**		25 (52)	
		≤30 minutes	14 (29)	
		31-90 minutes	9 (19)	
		91 minutes-4.5 hours	2 (4)	
	**Duration (weeks)**		16 (33)	
		1-4	9 (19)	
		≥4	7 (15)	
	Duration not specified		12 (25)	
**Theoretical models^a^**				
	No model mentioned		16 (33)	
	Motivational interviewing		16 (33)	
	Cognitive behavioral therapy		6 (13)	
	Transtheoretical model		4 (8)	
	Theory of planned behavior		4 (8)	
**Intervention delivery**				
	**Outreach (clients had not sought help)**		32 (67)	
		Social media	9 (19)	
		Clinician or health service referral	8 (17)	
		Print marketing (flyers or brochures)	6 (13)	
		Trial panel (eg, Amazon Mechanical Turk)	6 (13)	
		Email (student email, newsletters, or from the electronic medical record portal)	5 (10)	
		Digital marketing (web-based advertisements or media)	5 (10)	
		Postal screening questionnaire	4 (8)	
		General practitioner waiting room	2 (4)	
		Events (community events or school workshops)	2 (4)	
	**Before target treatment (clients had already sought help)**		12 (25)	
		Before first use or session	6 (13)	
		On waiting list for treatment or assessment	3 (6)	
		During intake	2 (4)	
		Before intake	1 (2)	
	**Self-selected (clients were interested in help)**		3 (6)	
		Downloaded screening app	1 (2)	
		Via e-mental health portal	1 (2)	
		Via referral website for clinic	1 (2)	
	Unclear		1 (2)	

^a^Other models used in only 1 or 2 studies: health belief model, acceptance and commitment therapy, self-determination theory, unified theory of acceptance and use of technology, screening brief intervention and referral to treatment, motivational enhancement therapy, theory of reasoned action, and extended parallel process model.

### Intervention Components

The 48 interventions examined in the included studies all comprised several different topics and tools, which we refer to as components. The most prevalent component was general psychoeducation (40/48, 83%), followed by symptom assessment (23/48, 48%) and information on various treatment options (21/48, 44%). Refer Table 4 for a list of the 14 most common components. Other components included in <4 studies were self-monitoring, data security information, personal strengths, therapeutic alliance and roles in therapy, acceptance, imaginative exercises (eg, imagining ideal life or future with or without treatment), MI techniques (eg, importance and readiness rulers), and information about the effectiveness or advantages of a specific target treatment.

Identifying components that showed evidence of effectiveness was difficult owing to the variety of interventions and components covered in this review, as well as the diversity in the outcomes of the experimental studies (see the *Feasibility* section for a closer look at these outcomes). Some studies that compared 2 interventions with different components found no differences among the outcomes of these interventions [64,86]; however, other studies found the opposite (ie, different components in similarly delivered interventions resulted in significantly different outcomes [65,67,90]). In 2 studies aimed at social anxiety [65,90], the addition of components such as the pro-con list, goal setting, values, and planning led to significantly greater engagement with further treatment or help-seeking behaviors than did interventions without these components. Interestingly, another study found that the effectiveness of the components depended on the condition in question; in this case, providing tailored feedback on screening was detrimental when it came to social anxiety but not depression [75].

**Table 4:** Components used in the included interventions.

Component	Description	Frequency, n (%)
Psychoeducation	Information about condition, symptoms, risks, prevalence, treatment benefits, recovery chances, and myth busting	40 (83)
Assessments	Self-administered assessments of symptoms or behavior	23 (48)
Treatment options	Information about potential treatment options	21 (44)
Assessment feedback	Tailored or generic feedback on assessments; for example, severity relevant to the general population	18 (38)
Referral information	Direct contact information or guidance for further treatment	17 (35)
Testimonials	Videos or written stories from people with similar issues or from those who have been through treatment	16 (33)
Expectation management	Guiding expectations on treatment or help seeking and expectation setting	16 (33)
Pro-con list	Cost-benefit analysis of change, treatment, or help seeking	15 (31)
Coping skills	Cognitive behavioral therapy skills (eg, cognitive restructuring or behavioral activation), relaxation, mindfulness, and emotion regulation	10 (21)
Planning	Planning for change or treatment or planning for overcoming obstacles to change or treatment (implementation intentions)	8 (17)
Goal setting	Personal goals, life goals, and treatment goals	8 (17)
Values	Using values to develop discrepancy between ideal and actual self	5 (10)
Self-efficacy	Building belief in ability to change, self-esteem, and positive self-affirmations	4 (8)
Problem-solving	Identifying problems, brainstorming solutions, and solution planning	4 (8)

### Design Processes

Only 18 of the 45 (40%) included papers discussed how the intervention was designed or developed. Of these 18 studies, only 4 (22%) mentioned the design approach: a study used a user-centered design [59], one used a person-based approach [58], another used a participatory design [40], and the final study used a participatory design, ethnography, and co-design [60]. In terms of the design methods used in the development of the interventions, 9 (20%) studies included consultation with experts or input from expert groups [56,58-60,63,77,84,89,91], 5 (11%) studies used expert or user surveys [50,55,58,60,84], 4 (22%) conducted focus groups with users [40,59,60,78], and 3 (7%) conducted interviews with either users or experts [55,59,60]. A total of 2 (4%) studies reported using working groups comprising users with lived experience and experts to cocreate the intervention [60,77], and 2 (4%) used expert-only working groups [56,63]. A total of 12 (27%) studies reported conducting user testing of their interventions, usually with an iterative process of implementing feedback. A study conducted feasibility testing with clinicians [56].

### Feasibility

To better understand the effectiveness of the included interventions, we took the controlled studies (24/45, 53%) and charted their outcomes (Table 5). The outcome measures across the studies were diverse and ranged from behavior to intentions and attitudes toward further treatment. Other associated factors such as symptom improvement, mental health literacy, stigma, and acceptance were also used as proxy measures to infer subsequent behavior or action. Controls included treatment as usual, wait-list, no intervention, intervention control, and attention controls. For the attention controls, we distinguished between nonspecific treatment component controls and specific treatment component controls [92].

Of the 16 studies that measured actual behavior or action (eg, engagement with target treatment or help-seeking behavior), 7 (44%) showed evidence of intervention effectiveness compared with controls [63,65,66,74,87,89,90]. However, these results should be considered in the context of other findings in the studies. For example, a study of an MI-based program aimed at preparing clients for web-based CBT found that clients in the intervention group (IG) spent longer time using the target treatment than those in the control group (CG), but their symptoms were actually worse after the treatment [89]. The participants in this study were highly motivated to engage in treatment at screening, which should also be noted along with the results.

A further 7 studies found no differences between controls and interventions in terms of behavior [54,62,64,67,68,78,86]; however, the other results in these studies provide vital qualifying information. For example, Denison-Day et al [54] offered the intervention to clients in the IG but allowed for natural uptake, meaning that only 34% of the IG actually used the intervention. Hence, no differences were found among groups when 98% of those who actually used the intervention engaged in further treatment. The type of control also had a considerable impact on whether the interventions were found to be “effective” (eg, Brunette et al [86] found no differences among groups, but both groups were given interventions, and both had high subsequent use of target treatment). In some studies with no intervention controls, both groups were found to exhibit high adherence to the target treatment [68].

A total of 2 studies indicated worse behavioral outcomes for the IG compared with the CG [75,80]. Again, the control and other results need to be considered; in the study by Krampe et al [80], the “treatment-as-usual” CG received both the digital intervention and MI-based FTF psychotherapy sessions, whereas the IG received the digital intervention alone, and their results showed that the digital intervention was comparable with the FTF control for those with high readiness to change scores [80]. In the study by Batterham et al [75], the IG received tailored feedback after screening based on symptom severity, whereas the CG received generic, untailored feedback. For clients with social anxiety, tailored feedback led to lower treatment use and intentions to seek help rather than generic advice. Study attrition was lower in the IG than in the CG; however, this is another factor to consider along with these results [75].

Considering the other variables measured in these studies, the findings are mixed. Some indicated that the interventions increased help-seeking intentions [72,83], whereas others showed no effect on intentions [64,71,79], despite their effectiveness in improving attitudes toward treatment or motivation to change. Some indicated improved symptoms [62,71,78], whereas others reported reduced stigma or improved literacy [14,52,72,79]. In addition, all the pre-post studies in the review found that their interventions either reduced client symptoms [53,73] or increased client interest in further treatment [51,73], and all the observational studies in the sample indicated positively skewed effects of their interventions on help-seeking actions, behaviors, or intentions [49,57,69,76,77,82,84].

No obvious patterns were observed among intervention format, support level, duration, components (see the *Intervention Components* section), condition, target treatment or intervention delivery, and whether interventions were effective. Several studies that compared interactive and noninteractive interventions suggested that interactivity is important for effectiveness [62,87,90]; however, the opposite result was also found [86].

**Table 5:** Outcomes of the controlled studies in the sample (standardized measures are abbreviated).

Study	Study design	Control	Sample size, N	Intervention	Measures	Significant outcomes
Olson et al [56]	Historically controlled	TAU^a^	163	Screening tool	Acceptance and quality of physician appointment survey; qualitative physician feedback	IG^b^ more likely to discuss alcohol and tobacco use with physician but not mood disorders. IG increased acceptance of subsequent physician appointment
Yoon et al [91]	Historically controlled	TAU	301	Screening tool	Screen for unhealthy drinking behaviors and alcohol use disorders; motivation to change and referral interest survey; acceptance survey	CG^c^ used to compare response rate only (responses were comparable). Only 16% of the IG had unhealthy drinking habits. Of these, 14% were interested in further help, and 40% would cut back on their own
Bommelé et al [78]	NRCT^d^	NTCC^e^	757	WP^f^	PO^g^: receptivity to information, motivation to change, self-efficacy and referral interest survey; SO^h^: cigarettes per day and quit attempts	IG more receptive to information than CG after the intervention but not at the 2-week or 2-month follow-up. IG had reduced smoking at all time points. No differences in quit attempts or referral
Brunette et al [66]	NRCT	Wait-list	41	WP	PO: treatment seeking and motivation to change survey (verified by medical records); SO: FTND^i^; 1 item from SCS^j^; ATS^k^	IG more likely to have taken action toward change than CG (eg, attempting to quit, meeting with a clinician to discuss, or start treatment)
Strassle et al [68]	RCT	No intervention	68	Video	PO: return for second session of TT^l^; SO: SCL-90^m^; IIP-32^n^; CASF-P^o^; therapist measures: GAF^p^; CASF-T^q^	No differences between IG and CG in adherence to TT, therapeutic alliance, or TT outcomes (all clients had high adherence to TT)
Ebert et al [14]	RCT	No intervention	128	Video	PO: acceptance survey; SO: expectations, social opinions, internet concerns, help-seeking attitudes, and web-based therapy literacy survey	IG had higher acceptance, expectations, and literacy and lesser internet concerns than CG. No differences in social opinions or help-seeking attitudes
Ebert et al [83]	RCT	No intervention	1374	Screening tool	PO: intention to seek help survey; moderators: CIDIS^r^; AUDIT^s^; CSSR^t^; SITBI^u^; subjective health, lifetime and current treatment use, intention to use mental health services, barriers to treatment use, and readiness to change survey	IG had higher intentions to seek help than CG. Intervention was more effective for those with panic disorder and worse physical health and those who were nonheterosexual. No effect of intervention for those in the action stage of change
Soucy et al [89]	RCT	No intervention	231	WP	PO: CQ^v^; TT lessons accessed; GAD-7^w^; PHQ-9^x^; SO: motivation to engage in TT survey; acceptance survey; K10^y^; SDS^z^	IG spent longer in TT than did CG. IG had higher anxiety and perceived disability at post-TT period than did CG. No differences in motivation or acceptance
Christensen et al [62]	RCT	NTCC	414	2 IGs: W^aa^ and WP	CES-D^ab^; help- and treatment-seeking survey	Both W and WP reduced depression symptoms compared with CG. W less likely to seek informal help than CG. WP more likely to use certain evidence-based treatments
Reis and Brown [63]	RCT	NTCC	125	Video	Therapist measure: TSQ^ac^	IG had lower dropout from TT than did CG
Costin et al [64]	RCT	NTCC	348	2 IGs: both automated emails and W	PO: AHSQ^ad^; informal help-seeking survey; SO: GHSQ^ae^; beliefs about help-seeking survey; depression and help-seeking literacy survey; CES-D; acceptance survey	No differences among IGs or between IGs and CG in help-seeking behavior, intentions, literacy, or depression symptoms. IGs had more positive beliefs about formal help than did CG
Johansen et al [67]	RCT	NTCC	105	2 IGs: WA^af^ video and EA^ag^ video	Acceptance survey; PANAS^ah^; WAI-S^ai^ (client and therapist); return for second session of TT	WA had higher negative affect and lower therapist-rated alliance than CG. No difference in client-rated alliance among IGs. No differences in adherence to TT between IGs and CG
Taylor-Rodgers and Batterham [72]	RCT	NTCC	67	WP	PO: A-Lit^aj^; D-Lit^ak^; LSS^al^; DSS^am^; GASS^an^; SOSS^ao^; ATSPPH-SF^ap^; GHSQ; SO: PHQ-9; GAD-7; acceptance and adherence survey	IG had increased anxiety literacy, help-seeking attitudes and intentions, and reduced depression stigma compared with CG. No differences in symptoms, acceptance, or adherence
Griffiths et al [79]	RCT	NTCC	83	WP	PO: GHSQ; SO: ATSPPH-SF; SA-Lit^aq^; SASS-I^ar^; perceived need for treatment and interest in TT; acceptance survey	IG had higher literacy, perceived need, and positive attitudes toward treatment than did CG. No differences in help-seeking intentions or stigma
King et al [74]	RCT	STCC	76	Screening tool and messaging	Perceived need for help and treatment use survey; 2 items from DDS^as^; readiness to access help survey	IG had higher readiness to access help and use treatment and lower stigma than did CG at the 2-month follow-up
Batterham et al [75]	RCT	STCC	2773	Screening tool	PO: AHSQ; SO: PHQ-9; SOPHS^at^ 2 items from GHSQ; AQoL-4D^au^; self-reported days out of role	IG had higher study attrition than did CG. For social anxiety, IG had lower treatment use and intentions to seek help than did CG, no differences found for depression
Peter et al [87]	RCT	STCC	805	2 IGs: screening tools—IM^av^ and NM^aw^	PO: choice between BBGS^ax^ and 3 items from GBQ^ay^; moderators: gambling history, psychological distress, and treatment interest survey	IM more likely to complete gambling screener than NM or CG
Titov et al [65]	RCT	Intervention control	108	2 IGs: WPs—Education and Education+Motivation	PO: SIAS^az^; SPS^ba^; SO: PHQ-9; K-10, SDS, and CEQ^bb^; literacy and motivation to change survey; time spent, log-ins, and homework downloads of TT	Education+Motivation had higher use of TT than Education. No differences in TT outcomes or acceptability. No differences in motivation to change
Tobias et al [90]	RCT	Intervention control	267	2 IGs: WPs—Education and Education+Motivation	Motivation for individual treatment steps, attitudes toward and intentions to seek treatment, perceived ability to engage in treatment seeking, and treatment use survey; CSQ-8^bc^	Education+Motivation had improved treatment-seeking attitudes and behaviors, compared with Education. Both groups improved on all outcomes
Brunette et al [86]	RCT	Intervention control	162	2 IGs: WPs—IWP^bd^ and DEP^be^	PO: treatment use (verified by medical records); SO: expired carbon monoxide; TFB^bf^ (quit attempts); PUEUS^bg^	No differences between IWP and DEP in TT use, quit attempts, or abstinence (both groups had high use of TT)
Denison-Day et al [54]	RCT	TAU	313	WP	PO: attendance at initial assessment appointment; SO: use of TT, acceptance, and motivation (interview)	No differences between IG and CG in attendance at initial appointment. Only 34% of the IG used the intervention, and of these, 98% attended the appointment
Krampe et al [80]	RCT	TAU	220	Screening tool	PO: treatment use; SO: URICA^bh^; BSI-GSI^bi^	IG had lower treatment use and worse symptoms than CG. IG and CG were comparable for those with high readiness to change scores
Keller et al [52]	RCT	Wait-list	320	3 IGs: videos—7 minutes, 13 minutes, and 17 minutes	SSOSH^bj^; stigma survey	Only the 17-minute IG reduced stigma compared with CG
Hötzel et al [71]	RCT	Wait-list	212	WP	PO: SOCQ-ED^bk^; SO: P-CED^bl^; SES^bm^; RSES^bn^; EDE-Q^bo^	IG had higher motivation to change, self-esteem, and symptom improvement than CG. No differences in motivation to begin treatment

^a^TAU: treatment as usual.

^b^IG: intervention group.

^c^CG: control group.

^d^NRCT: nonrandomized controlled trial.

^e^NTCC: nonspecific treatment component controls.

^f^WP: web-based program.

^g^PO: primary outcomes.

^h^SO: secondary outcomes.

^i^FTND: Fagerström test for nicotine dependence.

^j^SCS: Stage of Change Scale.

^k^ATS: Attitudes Toward Smoking Scale

^l^TT: target treatment.

^m^SCL-90: Symptom Checklist-90-Revised.

^n^IIP-32: Inventory of Interpersonal problems-32.

^o^CASF-P: Combined Alliance Short Form-Patient version.

^p^GAF: Global Assessment of Functioning Scale.

^q^CASF-T: Combined Alliance Short Form-Therapist version.

^r^CIDIS: Composite International Diagnostic Interview Screening Scales.

^s^AUDIT: Alcohol Use Disorders Identification Test.

^t^CSSR: Columbia Suicidal Severity Rating Scale.

^u^SITBI: Self Injurious Thoughts and Behaviors Interview.

^v^CQ: Change Questionnaire.

^w^GAD-7: Generalized Anxiety Disorder 7-item.

^x^PHQ-9: Patient Health Questionnaire 9-item.

^y^K-10: Kessler 10-item.

^z^SDS: Sheehan Disability Scales.

^aa^W: website.

^ab^CES-D: Centre for Epidemiological Studies Depression Scale.

^ac^TSQ: Termination Status Questionnaire.

^ad^AHSQ: Actual Help Seeking Questionnaire.

^ae^GHSQ: General Help Seeking Questionnaire.

^af^WA: working alliance.

^ag^EA: experimental acceptance.

^ah^PANAS: Positive and Negative Affect Schedule.

^ai^WAI-S: Working Alliance Inventory.

^aj^A-Lit: Anxiety Literacy Scale.

^ak^D-Lit: Depression Literacy Scale.

^al^LSS: Literacy of Suicide Scale.

^am^DSS: Depression Stigma Scale.

^an^GASS: Generalised Anxiety Stigma Scale.

^ao^SOSS: Stigma of Suicide Scale short form.

^ap^ATSPPH-SF: Attitudes Toward Seeking Professional Help Short Form Scale.

^aq^SA-Lit: Social Anxiety Literacy Questionnaire.

^ar^SASS-I: Social Anxiety Stigma Scale.

^as^DDS: Discrimination-Devaluation Scale.

^at^SOPHS: Social Phobia Screener.

^au^AQoL-4D: Assessment of Quality of Life.

^av^IM: interactive message.

^aw^NM: noninteractive message.

^ax^BBGS: Brief Biosocial Gambling Screen.

^ay^GBQ: Gamblers’ Beliefs Questionnaire.

^az^SIAS: Social Interaction Anxiety Scale.

^ba^SPS: Social Phobia Scale.

^bb^CEQ: Credibility/Expectancy Questionnaire.

^bc^CSQ-8: Client Satisfaction Questionnaire.

^bd^IWP: interactive web-based program.

^be^DEP: digital education pamphlet.

^bf^TFB: Timeline Follow-Back method.

^bg^PUEUS: Perceived Usefulness and Ease of Use Scale.

^bh^URICA: University of Rhode Island Change Assessment.

^bi^BSI-GSI: Global Severity Index of the Brief Symptom Inventory.

^bj^SSOSH: Self-Stigma of Seeking Help Scale.

^bk^SOCQ-ED: Stages of Change Questionnaire for Eating Disorders.

^bl^P-CED: Pros and Cons of Eating Disorders Scale.

^bm^SES: Self-Efficacy Scale.

^bn^RSES: Rosenberg Self-Esteem Scale.

^bo^EDE-Q: eating disorder symptomatology.

## Discussion

### Principal Findings

This scoping review explores digital interventions to enhance readiness for psychological therapy. These interventions are delivered most often as unsupported web-based programs designed for once-off use that takes <90 minutes. They are used to prepare clients for specific therapies or, more generally, to enhance readiness for professional treatment; they are provided to clients either via outreach methods for those who have not sought help, or they are inserted into the care pathway before the main treatment for those who have already reached out. Thus, these interventions appear to cater to clients across multiple stages of change, from those in precontemplation, who are not yet aware that they need help, to those in the preparation stage, who are taking initial steps toward change.

What is the most apparent from this review is the substantial variation not only in the types of digital readiness interventions that have been used but also in their development, delivery, and evaluation. When it comes to the feasibility of digitally delivering interventions, the included studies indicate that there is potential in this area. The current state of the literature, however, does not yet support the possibility of determining which components or types of interventions are effective or not effective; this is a complex undertaking with multiple factors to consider. For example, in some contexts, interactivity appears to be an important aspect of these interventions, which makes sense when considering the conversational nature of traditional FTF MI. However, many simple, noninteractive interventions such as videos and advertisements were also effective at improving variables related to further treatment seeking or engagement. Despite the variability among the studies included in this review, several common topics emerged: *tailoring to the stage of change, intervention pathways*, *risk*, and *evaluation*.

### Tailoring to the Stage of Change

The existing literature indicates the effectiveness of tailoring psychosocial interventions to clients’ stages of change [29]. Several studies in our review involved tailoring to the stage of change [58,69,73,84]. In 2 studies, tailoring involved a simple 2-way split, with different content for those who were interested in further treatment and those not interested [69,73]. In one of these studies, clients who were not yet interested in further treatment were given CBT coping techniques as a way to show them how treatment works and how effective it can be, rather than simply telling them this [73]. When clients are highly motivated, tailored interventions tend to focus on the practical aspects of engaging with further treatment (ie, choosing the right treatment, setting expectations, and planning).

The effective identification of a client’s stage of change is a significant aspect of tailoring. This can be done by asking simple binary questions, such as those in the aforementioned studies (eg, Are you interested in treatment?) or more formally with readiness measures such as the General Help Seeking Questionnaire [93], Stage of Change Scale [94], or University of Rhode Island Change Assessment [95]. One interesting website intervention used the stages of change to frame the headings of the main website navigation (ie, “Do I have a problem?” “Should I get help?” “I want and need help?” “I have tried to get help”), giving the client agency in self-selecting their own stage and thus controlling and tailoring their own journey [84]. Outside the stages of change, information-based interventions can be tailored to the client’s personal circumstances and needs at a broader level. For example, Dreier et al [50] provided suicide stigma interventions that were modified depending on whether clients had a suicide attempt in the past, had suicidal thoughts, had lost a close person by suicide, were fearing the loss of a close person by suicide, or were interested in the topic in general.

In all, 2 studies in this review illustrate the importance of effective stage identification and tailoring, with findings indicating negative or no effects of their inventions on those who already had high motivation or intentions to seek help [83,89]. Previous research also demonstrated that FTF MI is most beneficial for those who are not already motivated or engaged in treatment [96]. However, in contrast to this, Krampe et al [80] found that their brief digital intervention was as effective as FTF MI–based psychotherapy but only for clients who were already motivated. Tailored digital readiness interventions have the potential to bridge the divide between client and treatment, providing light-touch interactions for those who are already motivated as well as more detailed programs for those in earlier stages of change. For clients, these interventions could serve as stepping stones between information gathering and formal treatment, with layered interactions that support individuals on their journey through the stages of change [77].

### Intervention Pathways

The implementation of digital readiness interventions involves both onboarding (ie, the uptake of the intervention itself) and off-boarding (ie, the link between the intervention and further treatment). In terms of onboarding, the first point of contact and framing of digital readiness interventions are crucial, as uptake issues can drastically impact their effectiveness in the real world. Denison-Day et al [54] found that although their intervention was highly effective for those who used it, only 34% of the IG actually used it (they offered participants the intervention but allowed for natural uptake). They noted that simply offering new interventions to address the problem of target treatment engagement may not be enough, and approaches focused on low engagement may need to be considered even earlier in the treatment pathway. An interesting aspect of their intervention (further detailed in the development process paper by Muir et al [58]) was that instead of aiming to prepare clients for the full extent of treatment, they framed it as preparation for the initial assessment appointment only. This removed some of the overwhelming aspects of thinking about full “recovery” and instead allowed clients to take their treatment journey 1 step at a time [58]. How the first step on a client’s journey is presented and by whom could have an impact on the client’s subsequent progress toward change.

Several studies included in this review were conducted in health care settings, where client trust has already been established [59,80,91]. Embedding readiness interventions within existing pathways, such as routine screening, general practitioner waiting rooms, or treatment waiting lists, can draw on this trust and help the client gain direct access to appropriate services. Regarding off-boarding, many studies in this review noted that access to the target treatment needs to be provided in a timely manner following the readiness intervention, as motivation wanes over time [66,74,78,82,86,90]. Moessner et al [76] included clinician monitoring of client deterioration as part of their intervention, allowing clients to take their time to become ready for treatment, while still being supported. In the intervention developed by Brown et al [55], the first session of the target treatment immediately followed the readiness intervention (if the client wanted to proceed), making the most of their motivation and removing any lag time between the interventions. Where digital readiness interventions fit within the wider context of client pathways appears to be an important consideration for both their development and evaluation.

### Risk

An important aspect that surfaced while reviewing these studies was the potential risk of readiness interventions impairing treatment engagement, reducing help seeking, worsening symptoms, and increasing self-stigma. Batterham et al [75] found that tailored feedback on screening reduced help seeking for individuals with social anxiety compared with a control that was just generic information; the directive nature of this feedback may have come across as particularly confrontational to clients experiencing difficult emotions centered on their interactions with others. Similarly, Johansen et al [67] found that a video providing information on the working alliance between the client and therapist led to more negative emotions for the client and no improvement in working alliance ratings. Information designed simply to “prepare” clients for what is to come can potentially lead to negative emotions and apprehension, which can in turn affect readiness for treatment.

Stigma adds another layer of complexity to the help-seeking and treatment readiness process; Keller et al [52] found that informational videos on suicide prevention increased empathy, while simultaneously decreasing help seeking. Previous research shows that different types of stigma (eg, public stigma vs internalized self-stigma [97]) affect help seeking in different ways [98]. How we interpret the experiences and emotions of other people is distinct from how we perceive our own internal states. When addressing stigma with a digital readiness intervention, care should be taken as to which types of stigma are being targeted and the intricate relationships among them. Furthermore, stigma is not only complex, layered, and subjective, but even the act of measuring it can reproduce or reinforce stigmatizing attitudes [50]. Individual emotional responses to engaging in treatment for mental health difficulties are sensitive and differ from person to person; a delicate, cautious approach is clearly needed when developing and implementing readiness interventions.

### Evaluation

The final discussion concerns the evaluation of readiness interventions and issues when conducting research in such a sensitive area. Several studies in this review found that clients in the control arms improved as much as those in the intervention arms [64,68,86]. Considering the large battery of measures used in several studies and the fact that screening was a core component in many of the included interventions, it is difficult to separate the effects of the interventions themselves from the overall effects of trial participation. Although this is often the case with research trials, the specific light-touch, preparatory nature of these interventions makes them more susceptible to this reactivity. Perhaps, in many cases, being included in a trial focused on help seeking constitutes a readiness intervention in itself.

In addition, the trial design has a significant influence on the “effectiveness” of a given intervention. Constrained processes that force engagement with an intervention may provide rigor in intervention effects but have little ecological validity. There is also potentially greater baseline motivation among people who are prepared to participate in clinical trials than among the general population [65]. The real-world uptake of digital readiness interventions is key to their effectiveness. Naturalistic studies could therefore be a more useful method of understanding how these interventions would function in practice.

Another aspect of evaluation involves the chosen research methodology, which not only has a fundamental impact on the outcomes of the study but also on how we come to understand complex social constructs such as stigma, motivation, and the stages of change. Using quantitative measures to isolate and examine phenomena such as attitudes and emotions is limited because these experiences are highly subjective and contextual [52]; we miss vital information when we detach these occurrences from what gives them meaning. Considering that only one-fourth of the studies in this review included qualitative data collection, there exists a significant gap in our understanding of the nuances of this process at the individual level. Furthermore, many of the studies in this review used proxy measures, such as intentions and attitudes, to infer potential future action although the attitude-intention-behavior models that underpin these inferences have been contested in research across several fields [99-101]. This review suggests that the measurement of digital readiness interventions requires careful consideration because of the many intricacies involved.

### Limitations

There are several limitations to this study. First, we did not include help seeking as a search term (we decided to focus our search on the more general areas of readiness, preparation, and motivation); therefore, our coverage of help-seeking interventions was not comprehensive. Furthermore, our digital-only inclusion criteria excluded some interesting interventions that could easily be reproduced digitally (eg, a postal survey on implementation intentions [102] and an educational handout about the dose-effect relationship of therapy and expectation setting around treatment length [103]).

### Implications for Research

Given the inconclusive nature of findings presented here, further research is needed to enhance our knowledge and shape the field of digital readiness interventions for psychological therapy. In-depth qualitative research is crucial to understanding individual differences in emotional responses to readiness interventions and how constructs such as self-stigma affect motivation. Longitudinal research could also provide insights into individual trajectories through the stages of change because the process of becoming ready for treatment can be a long-term one, involving many layers and influences [76]. Recent phenomenological research indicates that change is perhaps a more continuous, internal, and holistic process than the TTM allows [104], and therefore, mapping the process of change in relation to readiness for mental health treatment would add depth to our theoretical foundations. Naturalistic effectiveness studies that attempt to reduce confounding trial effects and examine intervention implementation would help us to ground our knowledge in ecologically valid data and thus improve the practical application of digital readiness interventions. In addition, few studies in this review reported on how the interventions in question were developed or the design strategies used; this is important information for advancing the field and building best practices for future development. Finally, to further understand the different types of readiness interventions being used, future reviews could use more specific search terms (eg, help seeking, screening, and wait-list) to explore these areas in more detail. They could also include quality assessments in their charting process; however, the methodological issues discussed earlier would need to be further unraveled to enable a useful discussion of quality.

### Conclusions

Digital interventions to enhance readiness for psychological therapy are broad and varied. The interventions in question range from brief, simple videos and advertisements to supported web-based programs. They are used to help clients across multiple stages of change, from those in precontemplation who have not yet sought help to those already preparing to take action. Although these easily accessible digital approaches show potential as a means of preparing people for therapy and thus reducing the mental health treatment gap, they are not without risks. The complex nature of stigma, motivation, and individual emotional responses toward engaging in treatment for mental health difficulties suggests that a careful approach is needed when developing and measuring readiness interventions. The results of this review indicate that the implementation and uptake of these interventions are important elements to consider in design, delivery, and measurement and that further qualitative and longitudinal research is needed to deepen our knowledge of the process of change in relation to readiness for therapy. Overall, this review highlights the fact that the field of digital readiness interventions is an emerging one, and more research is needed in this area.

## Appendix

Multimedia Appendix 1 PRISMA-Scr checklist. PRISMA-Scr: Preferred Reporting Items for Systematic Reviews and Meta-Analyses Extension for Scoping Reviews.

## Abbreviations

CBT: cognitive behavioral therapy
CG: control group
FTF: face-to-face
IG: intervention group
MI: motivational interviewing
PRISMA-ScR: Preferred Reporting Items for Systematic Reviews and Meta-Analyses Extension for Scoping Reviews
TTM: transtheoretical model
